# Construction of competing endogenous RNA networks in systemic lupus erythematosus by integrated analysis

**DOI:** 10.3389/fmed.2024.1383186

**Published:** 2024-05-20

**Authors:** Juanjuan He, Yunfeng Dai, Jianwen Liu, He Lin, Fei Gao, Zhihan Chen, Yanfang Wu

**Affiliations:** ^1^Fujian Medical University Shengli Clinical Medical College, Fuzhou, China; ^2^Department of Rheumatology, Fujian Provincial Hospital, Fuzhou, China

**Keywords:** systemic lupus erythematosus, differentially expressed genes, competing endogenous RNAs, bioinformatics analysis, enrichment analysis

## Abstract

**Objective:**

Systemic lupus erythematosus (SLE) is a disease characterised by immune inflammation and damage to multiple organs. Recent investigations have linked competing endogenous RNAs (ceRNAs) to lupus. However, the exact mechanism through which the ceRNAs network affects SLE is still unclear. This study aims to investigate the regulatory functions of the ceRNAs network, which are important pathways that control the pathophysiological processes of SLE.

**Methods:**

CircRNA microarray for our tested assays were derived from bone marrow samples from three healthy individuals and three SLE patients in our hospital. The other sequencing data of circRNA, miRNA and mRNA were obtained from Gene Expression Omnibus (GEO) datasets. Using the limma package of R program, the differential expression of mRNA and miRNA in the GEO database was discovered. Then predicted miRNA-mRNA and circRNA-miRNA were established using miRMap, miRanda, miRDB, TargetScan, and miTarBase. CircRNA-miRNA-mRNA ceRNA network was constructed using Cytoscape, and hub genes were screened using a protein-protein interaction network. Immune infiltration analysis of the hub gene was also performed by CIBERSORT and GSEA.

**Results:**

230 overlapped circRNAs, 86 DEmiRNAs and 2083 DEmRNAs were identified in SLE patients as compared to healthy controls. We constructed a circRNA–miRNA–mRNA ceRNAs network contained 11 overlapped circRNAs, 9 miRNAs and 51 mRNAs. ESR1 and SIRT1 were the most frequently associated protein-protein interactions in the PPI network. KEGG analysis showed that DEGs was enriched in FoxO signaling pathway as well as lipids and atherosclerosis. We constructed a novel circRNA-miRNA-mRNA ceRNA network (HSA circ 0000345- HSA miR-22-3-P-ESR1/SIRT1) that may have a major impact on SLE.

**Conclusion:**

Through this bioinformatics and integrated analysis, we suggest a regulatory role for ceRNA network in the pathogenesis and treatment of SLE.

## Introduction

Systemic lupus erythematosus (SLE) is a complex autoimmune connective tissue disease that can lead to functional impairment of multiple organs and increased morbidity and mortality (1, 2). Many studies have confirmed the involvement of non-coding RNAs in the pathogenesis of lupus (3), while the exact pathogenesis is not fully understood. Thus, exploring the molecular features and mechanisms of non-coding RNA in SLE is crucial for better insight and treatment of the disease.

Competing endogenous RNAs (ceRNAs) are molecules of both coding and non-coding RNAs that can be targeted by the same miRNAs in the right context and can indirectly regulate each other by competing for them, leading to additional post-transcriptional regulatory layers where non-coding RNAs can find new meaning. miRNA-mediated interactions between different types of RNA molecules have been observed in many different contexts (4). Circular RNAs (circRNAs) lack 5′- and 3′-terminal poly (A) tails, making them less susceptible to nucleic acid exonuclease-driven degradation, resulting in a longer median half-life (5). Therefore, circRNAs are more likely to function as sponges and participate in the competing endogenous RNA (ceRNA) network (6). Researchers have experimentally tested instances of ceRNA crosstalk in a large number of situations and observed in normal and pathological contexts, demonstrating the widespread involvement of this mechanism in brain regenerative mechanisms (7), neuronal and muscle developmental processes (8) and cellular differentiation (9), as well as in diseases which highly complex gene regulatory circuits are most affected by perturbations. Researchers strive to model these regulatory networks to predict and understand how modifications alter the dynamic balance between molecules, leading to the onset of cancer and its progression, cardiovascular problems and neurodegenerative diseases, as well as other pathologies, such as those related to immune and autoimmune responses and degenerative physical conditions. Recently, several studies reported an association between ceRNA network and lupus (10, 11). The development of new computational tools that provide hypothetical predictions of ceRNA interactions facilitates insights into lupus pathogenesis and the discovery of novel therapeutic targets.

CircRNA, miRNA, and mRNA data can be obtained from the Gene Expression Omnibus (GEO) database (12), making it a valuable tool for biological discovery and data mining. Integrative ceRNA regulation circuits can be built to investigate more precise prognostic markers using these publicly available databases. In our study, we constructed ceRNAs networks of lupus by taking the intersection of our self tested circRNA microarray data with datasets from the GEO database. So far, such a study is unique.

In this research, we performed differential expression analysis using circRNA microarray of bone marrow samples from three healthy participants and three patients in our department and circRNA datasets in the GEO dataset. We then used online tools to identify the relationship between miRNA-circRNA and miRNA-mRNA to construct ceRNA network. By using enrichment function analysis, the biological significance of mRNA differential expression was determined. The protein-protein interaction (PPI) network (13) was used to search hub genes. Immune infiltration analysis of the hub gene was also performed by CIBERSORT and GSEA.

## Methods

### Bone marrow samples collection

Bone marrow samples were obtained from 3 healthy participants and 3 patients with systemic lupus erythematosus (SLE) at Fujian Provincial Hospital. All participants, both healthy and those with SLE, were of Han Chinese origin. The inclusion criteria required patients with untreated first-onset SLE and healthy subjects. SLE diagnosed according to the 1997 updated American College of Rheumatology (ACR) classification criteria (14). This research received approval from the Ethics Committee of the Fujian Provincial Committee. Informed consent was obtained from all participants.

### Data collection

We searched for SLE-associated aberrantly expressed RNA microarray data from the GEO database.^1^ The selection criteria were as follows: (a) The dataset included lupus patients and healthy controls. (b) Arrays contained at least 3 samples from patients and 3 samples from the normal group; (c) Uploaded data were available for analysis. Finally, GSE84655 (circRNA, 6 SLE vs. 3 control), GSE175840 (miRNA, 5 SLE vs. 5 control) and GSE175839 (mRNA, 5 SLE vs. 5 control) were included in the study. The downloaded files were calibrated, standardized, and log2 transformed using R software (15).

### Identification of the differentially expressed circRNAs, miRNAs and mRNAs

Batch effects were normalised using the sva package. Differential analysis was performed using the limma package. DEcircRNAs were identified using a threshold of |log2FC| over 1.2 and an adjusted *p*-value less than 0.05. Also, the ballgown package was employed to identify DEmiRNAs and DEmRNAs. The thresholds set were an adjusted *p*-value less than 0.05 and |log2FC| above 1.2 for DEmiRNAs and DEmRNAs.

### ceRNA network

The miRNAs corresponding to circRNAs were predicted by StarBase (16). To obtain the paired miRNA_mRNAs, miRMap (17), miRanda (18), miRDB (19), TargetScan (20), and miTarBase (21) were used to find possible targeted mRNAs of miRNA. Subsequently, the overlapping predictions between the two programs were considered effective target pairs. ceRNA networks were constructed in Cytoscape (version 3.7) (22).

### Gene function enrichment analysis

The database for annotation, visualization, and integrated discovery (DAVID) (23) was employed to determine the biological functions of DEGs. The biological process (BP), molecular function (MF), and cellular component (CC) were applied in GO enrichment analyses.^2^ The Kyoto Encyclopedia of Genes and Genomes (KEGG) serves as a database resource for comprehending biological systems and overarching functions (24). The cut-off criteria were the false discovery rate < 0.1 and adjusted *p*-value < 0.05.

### Formation of protein-protein interaction network and identification of hub genes

A PPI network of the DEmRNAs was established using the Search Tool for the Retrieval of Interacting Genes (STRING).^3^ Required Confidence (combined score) > 0.4 was selected as the threshold for PPI. The PPI network was visualized using Cytoscape. Molecular Complex Detection (MCODE) app of Cytoscape was used to determine hub genes.

### Immune infiltration analysis of hub-gene

CIBERSORT, an universal deconvolution algorithm, was employed to examine the immune cell subset proportions using RNA expression profiles (25). Herein, we obtained a matrix of 22 immune cell subsets in GSE50772 via the R package of CIBERSORT. A bar plot displayed the percent of each immune cell and compare infiltrating levels between SLE patients and controls using the “ggplot2 (v3.3.0)” package, and the “corrplot (v0.90)” package was utilized to depict the relationship of immune cell subsets. *p* < 0.05 was determined to be statistically significant.

### Gene set enrichment analysis

GSEA software (version 3.0) was obtained from the GSEA website.^4^ The Molecular Signatures Database^5^ was used to assess the pathways and molecular mechanisms involved in the hub genes. *p* < 0.05 was determined to be statistically significant.

## Result

### Identification of DEcircRNAs in SLE

**Figure 1:**
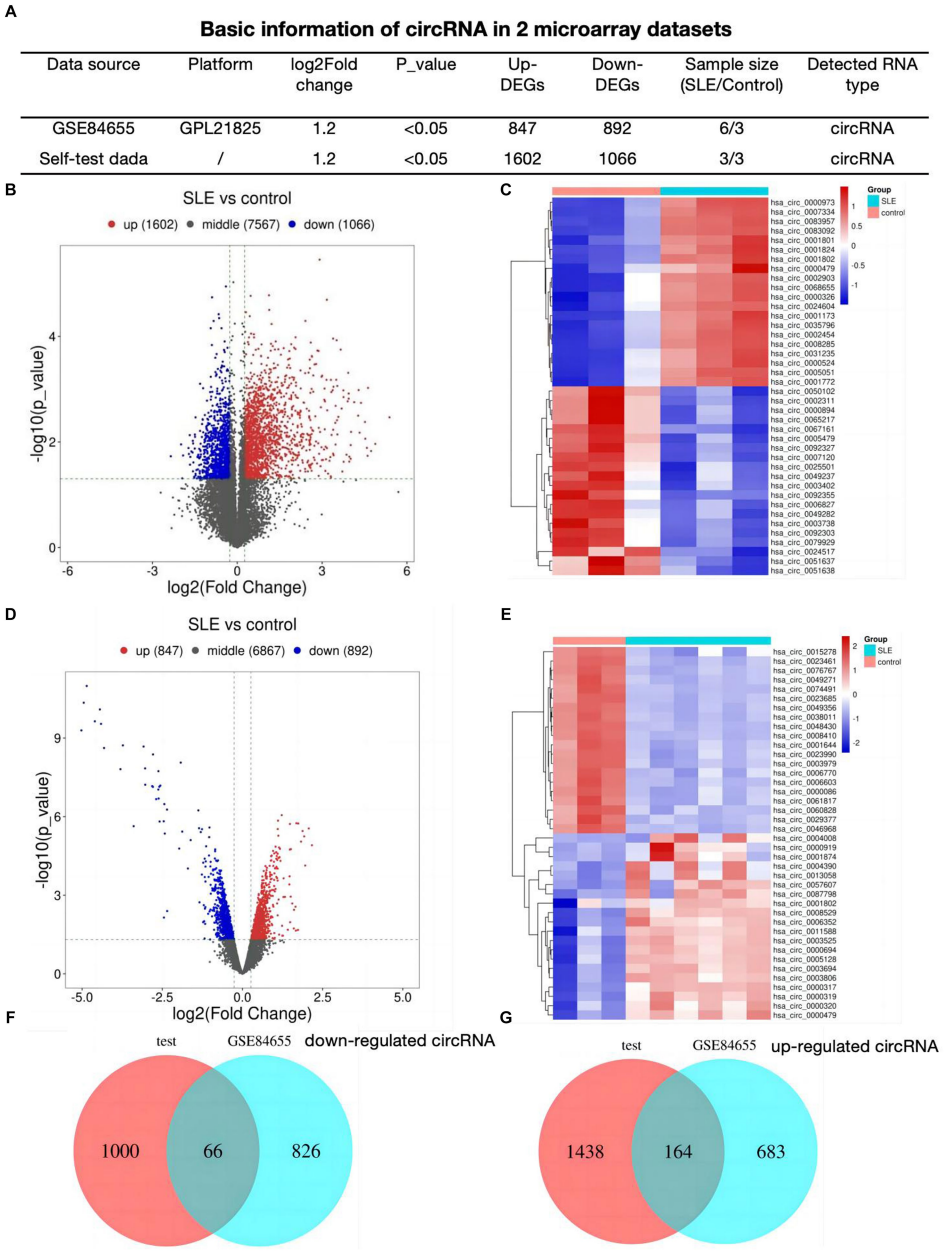
Identification of DEcircRNAs in SLE **(A)** Basic information of circRNA datasets. **(B)** Volcano plots for different expression of circRNAs of bone-marrow in SLE. **(C)** Heatmap plot showing the top 20 DEcircRNAs in our dataset. **(D)** Volcano plots for DEcircRNAs of GSE84655. **(E)** Heatmap plot showing the top 20 DEcircRNAs of GSE84655. **(F,G)** The Venn plots of DEcircRNAs of two datasets.

First, we analyzed 2 profiles of circRNAs including our self data and GSE84655, basic information was shown in Figure 1A. We detected 2,668 different expressed circRNAs (1,602 up-regulated, 1,066 down-regulated) in lupus samples using our tested bone marrow circRNA microarray (Figure 1B). Figure 1C showed the heatmap of top 20 differentially expressed circRNAs. To refine the identification further, a search for DEcircRNAs was conducted in GSE84655 and 1739 DEcircRNAs (847 up-regulated, 892 down-regulated) were found (Figure 1D). Subsequently, the top 20 circRNAs displaying the most significant variations were selected and are exhibited in Figure 1E. Ultimately, we integrated 230 overlapped circRNAs in SLE patients as compared to healthy controls (66 down regulated circRNAs Figure 1F and 164 up regulated Figure 1G).

### Determination of lupus-associated DEmRNAs and DEmiRNA

**Figure 2:**
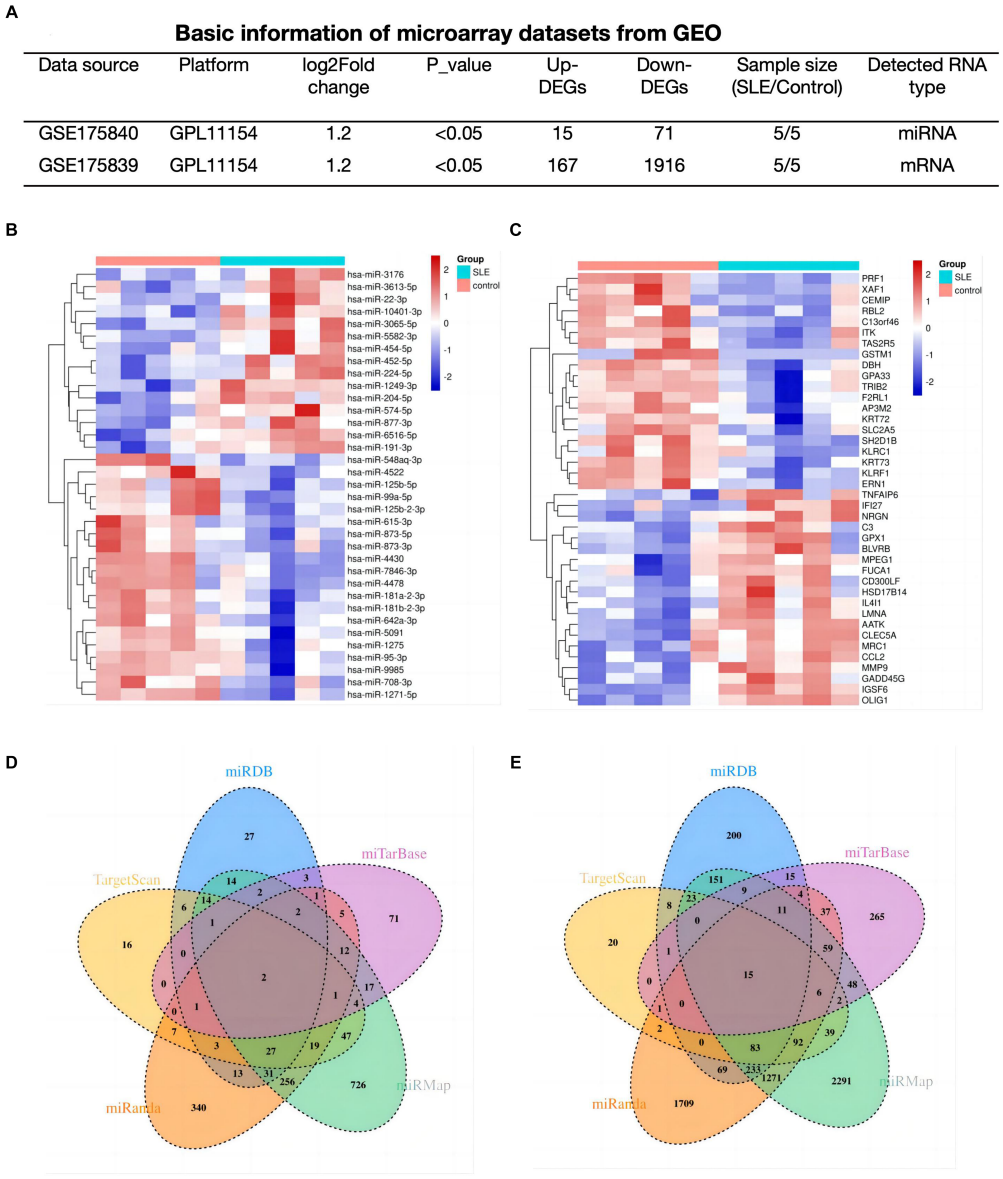
Determination of lupus-associated DEmRNAs and DEmiRNA. **(A)** Basic information of miRNAs and mRNA datasets. **(B,C)** Hotmap of the 20 top DEmiRNAs and DEmRNAs. **(D,E)** The Venn plots of predicted miRNAs based on DEmRNAs of GSE175839.

To explore the potential functions of mRNAs/miRNAs/circRNAs in SLE, The mRNA (GSE175839) and miRNA (GSE175840) datasets were further analyzed. In SLE group, 86 DEmiRNAs (15 up-regulated, 71 down-regulated), and 2083 DEmRNAs (167 up-regulated, 1916 down-regulated) were discovered based on predetermined thresholds (Figure 2A). The top 20 DEmiRNA and DEmRNAs revealed in Figures 2B,C.

In order to find out the targeted miRNAs corresponding to GSE175839, DEmRNAs were searched in the miRMap, miRanda, miRDB, TargetScan and miTarBase. Based on the principle that paired miRNA-mRNA presented at least four databases, we confirmed 34 pairs of down-regulated miRNA and up-regulated interacted mRNA (Figure 2D) and 115 pairs of up-regulated miRNA and down-interacted mRNA (Figure 2E).

### ceRNA network in SLE

Using the overlapped DEcircRNAs discovered in Figure 1, the 156 pairs of miRNAs that were down-regulated and the 1,180 pairs of miRNAs that were up-regulated were extracted from Starbase.

**Figure 3:**
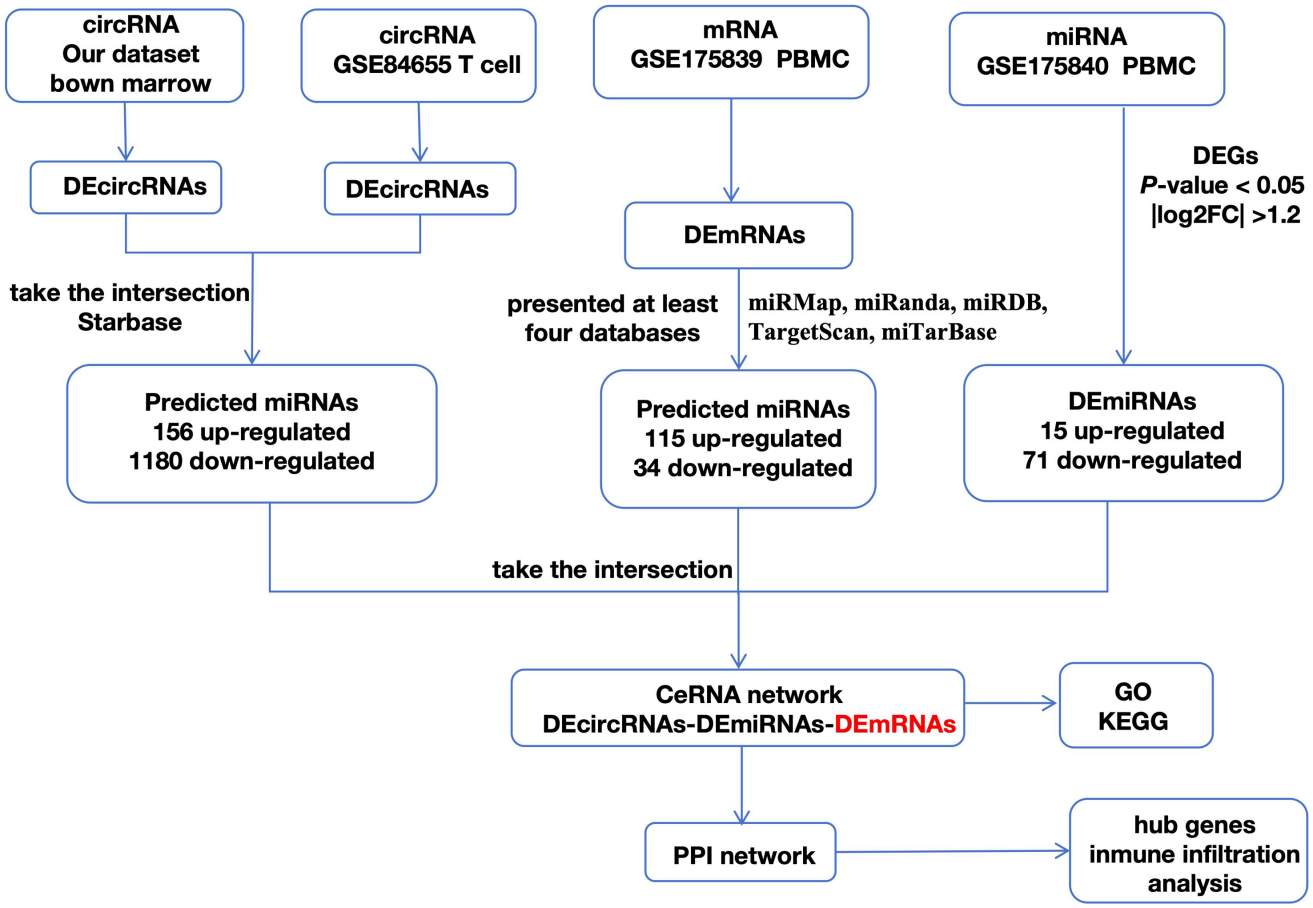
A flowchart of DEGs analysis.

**Figure 4:**
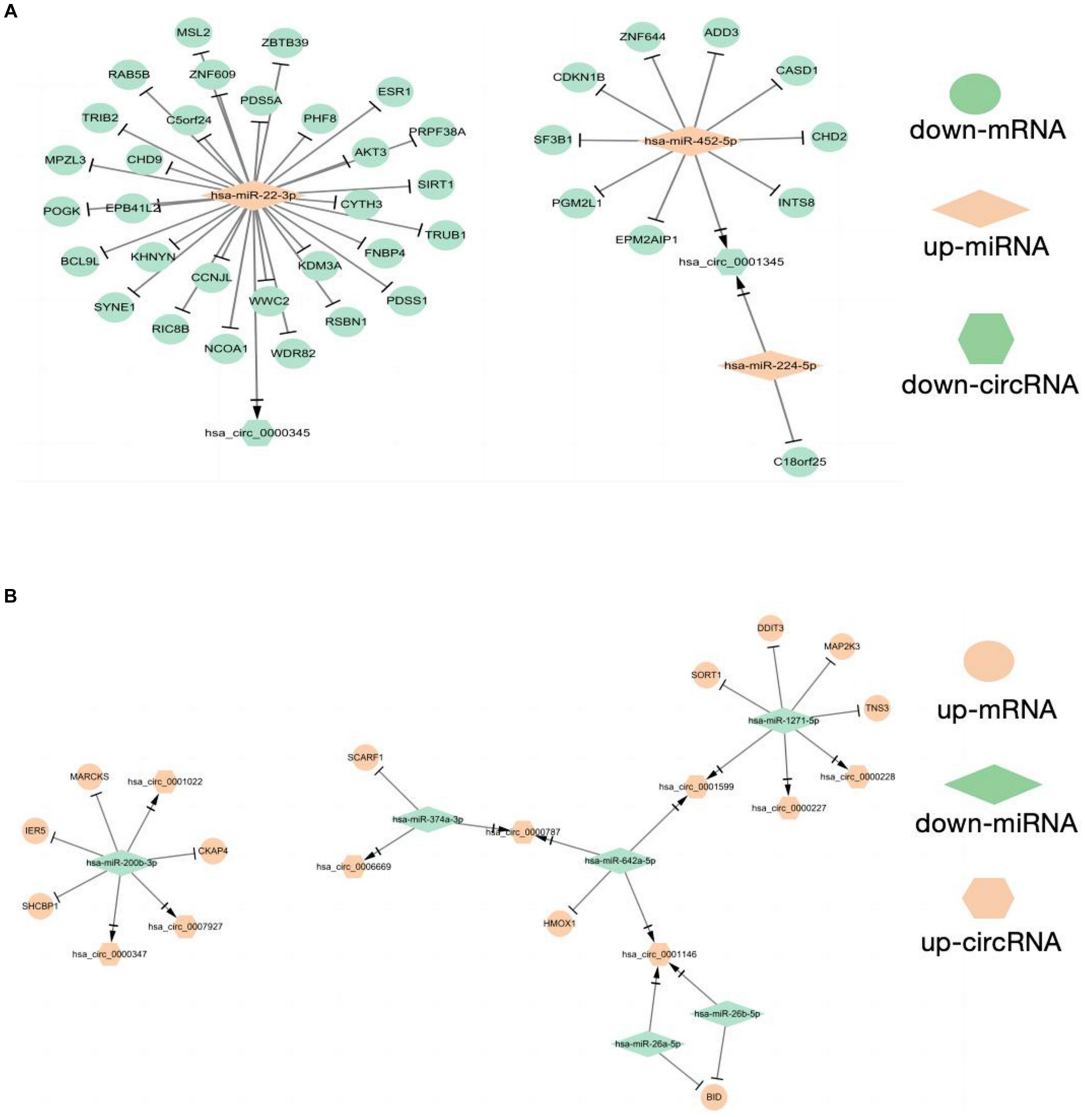
Construction of CeRNAs networks **(A)** CircRNA(down)-miRNA(up)-mRNA(down) regulatory network **(B)** CircRNA(up)-miRNA(down)-mRNA(up) regulatory network.

As shown in the flowchart (Figure 3), overlapped DEcircRNAs of (Figure 1) predicted cirRNA-miRNA, mRNA-miRNAs predicted by DEmRNAs of GSE175839 and the DEmiRNAs of GSE175840 were taken interacted, and identified the experimentally strongly supported SLE specific differentially expressed of the circRNA-miRNA-mRNA network. This network contained 11 circRNAs, 9 miRNAs and 51 mRNAs (Table 1). Then, we visualized these candidate genes and constructed the ceRNA network using cytoscapeV3.7 (Figure 4).

**Table 1:** Different expression of circRNAs-miRNAs-mRNAs in SLE.

DE circRNAs	Interacted DEmiRNAs	Targeted mRNAs
hsa_circ_0000345 hsa_circ_0001345	hsa-miR-22-3p hsa-miR-224-5p hsa-miR-452-5p	CHD9, CCNJL, PHF8, POGK, AKT3, BCL9L, C5orf24, ZNF609, MSL2, CYTH3, EPB41L2, ESR1, RIC8B, NCOA1, PDS5A, PDSS1, MPZL3, FNBP4, PRPF38A, RAB5B, ZBTB39, TRUB1, KDM3A, KHNYN, SYNE1, TRIB2, WDR82, WWC2, RSBN1, SIRT1, C18orf25, INTS8, EPM2AIP1, ZNF644, ADD3, PGM2L1, SF3B1, CHD2, CASD1, CDKN1B
hsa_circ_0000787 hsa_circ_0006669 hsa_circ_0001146 hsa_circ_0001022 hsa_circ_0007927 hsa_circ_0000347 hsa_circ_0000227 hsa_circ_0000228 hsa_circ_0001599	hsa-miR-642a-5p hsa-miR-374a-3p hsa-miR-26b-5p hsa-miR-26a-5p hsa-miR-200b-3p hsa-miR-1271-5p	MAP2K3, TNS3, DDIT3, SORT1, MARCKS, CKAP4, IER5, SHCBP1, BID, SCARF1, HMOX1

### Functional and pathway enrichment analysis for the targeted genes in the ceRNA network

**Figure 5:**
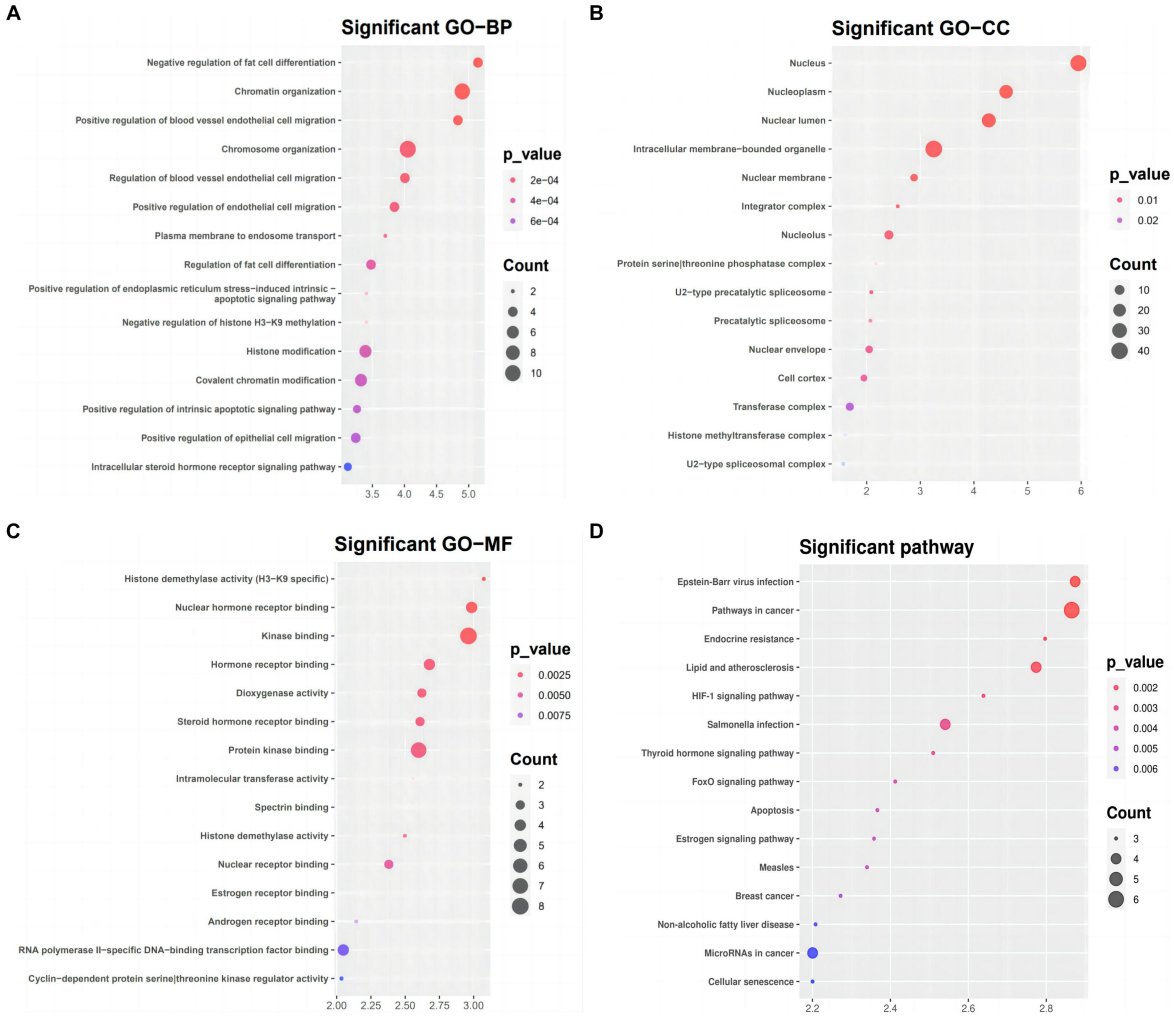
GO term and KEGG pathways enrichment analysis **(A)** Biological process (BP), **(B)** cellular component (CC), **(C)** molecular function (MF) of the GO annotation **(D)** KEGG pathways of DEmRNA.

We used DAVID to conduct functional and pathway enrichment analyses to gain more insight into the biological roles of the 51 DEmRNAs implicated in SLE. The top 15 KEGG pathways and enriched GO terms were exhibited in Figure 5. The GO analysis revealed that DEGs were primarily enriched in the following categories: “chromatin organization,” “chromosomal organization,” and “the negative regulation of fat cells” (BPs); “intracellular membrane-bounding organelle,” “nuclear lumen,” and “nucleotic” (CCs); and “kinase binding,” “protein kinase binding,” “RNA polymerase II-specific DNA binding transcription activator activity” (MFs). KEGG pathway analysis presented that DEGs of ceRNA network of SLE were enriched in malignancies, infection, Lipid and atherosclerosis, apoptosis, endocrine resistance, FoxO signaling, HIF-1 signaling, Estrogen signaling and thyroid hormone signaling (Figure 5D).

### Identification of hub genes

**Figure 6:**
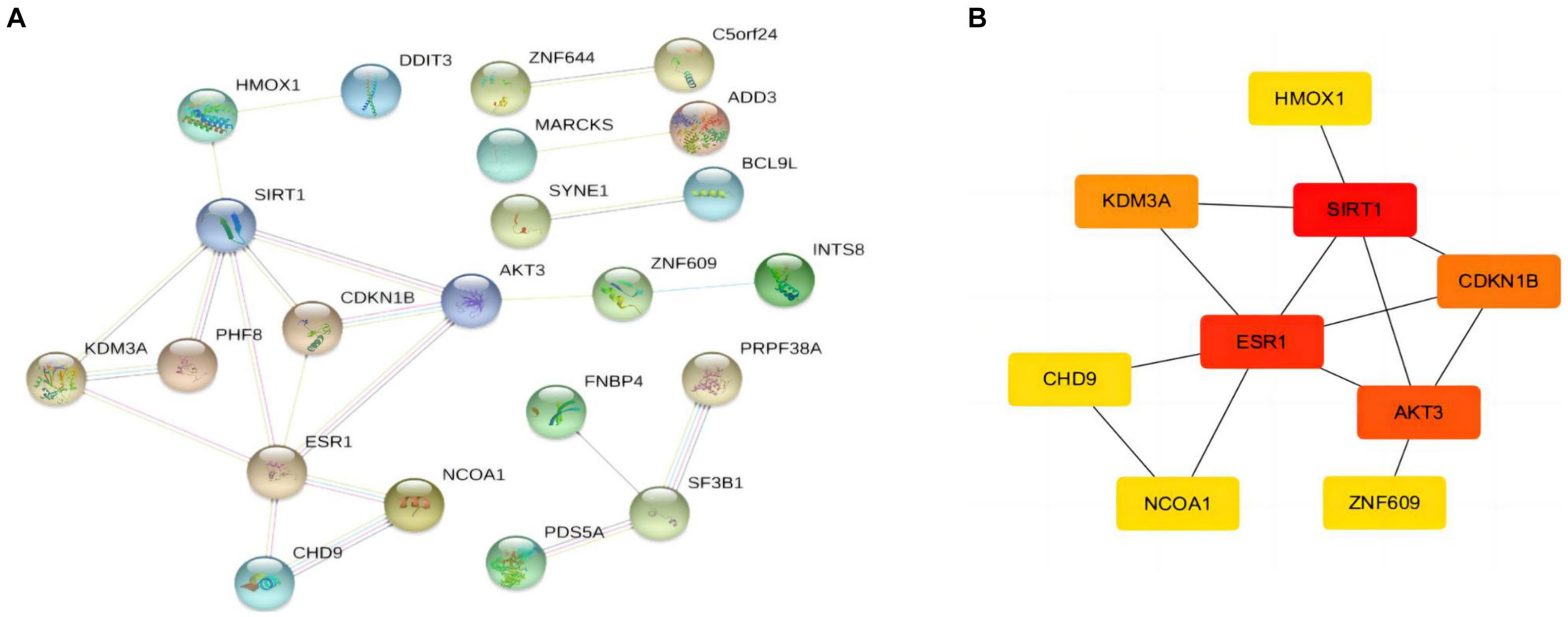
PPI network and hubgenes of targeted DEmRNAs in SLE **(A)** PPI network of DEmRNAs **(B)** The top 9 hubgenes analysed by Cytoscape.

After obtaining the target mRNA of ceRNA network in SLE, 51 DEmRNAs were imported into the PPI network composed of 51nodes and 22edges (Figure 6A). Following the identification of the vital functions of hub genes in the network, we used MOCDE plugin of cytoscape for further analysis, and 9 hub genes were explored (HMOX1, SIRT1, ESR1, AKT3, CDKN18, KDM3A, CHD9, NCOA1, ZNF609).

### Immune infiltration characteristics of hub gene SIRT1and ESR1

**Figure 7:**
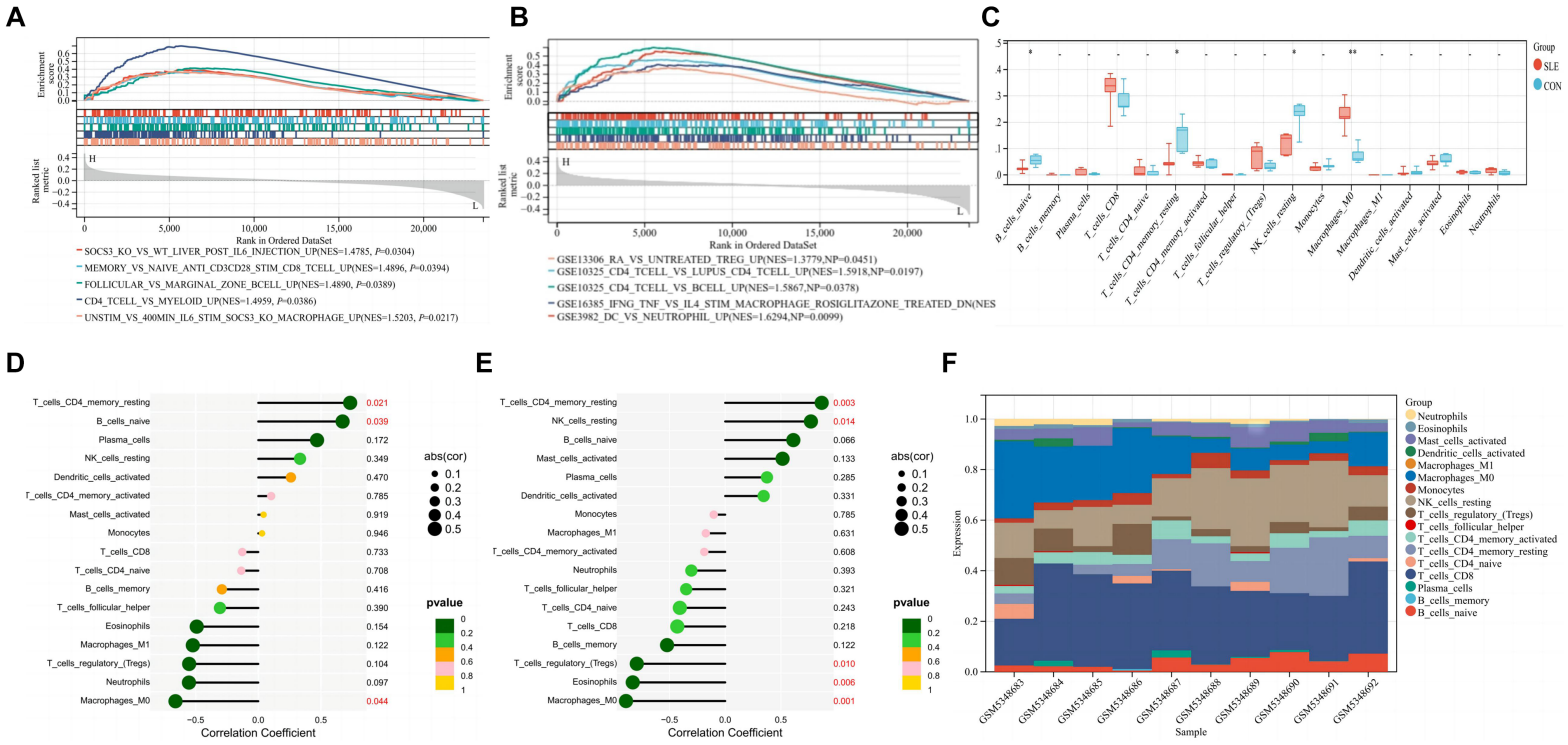
Immune infiltration characteristics between SLE patients and controls. **(A,B)** GSEA analysis for SIRT1 and ESR1 in SLE group. **(C)** Infiltrating difference of immune cells between SLE patients and controls in the box plot. **(D,E)** Spearman correlation between immune cell subsets for SIRT1 and ESR1. The color of dots denotes the P value. The size of dots denotes the strength of correlation. **(F)** The relative percent of immune cell subsets in a bar plot. Statistical significance at the level of * <0.05, ** <0.01, and *** <0.001. SLE: Systemic lupus erythematosus; CON: Controls.

When screening hub genes (Figure 6B), ESR1 and SIRT1 were the most frequently associated protein-protein interactions in the PPI network. To further investigate the mechanism of SIRT1 and ESR1 involvement in lupus pathogenesis, we analyzed SIRT1 and ESR1 using GSEA and also found that SIRT1 and ESR1 were involved in many biological processes of immune cells (Figures 7A,B), which had been demonstrated in SLE. Immune cell infiltration also confirmed the presence of statistically significant differences in CD4+ memory T cells, naive B cells and M0 Macrophages between SLE patients and controls (Figure 7C). Spearman correlation analysis was subsequently performed to demonstrate the relationship between DEOSRGs and various immune cell subsets. As shown in Figure 7D, SIRT1 was positively correlated with CD4+ memory T cells (r = 0.733, *p* = 0.021) and naive B cells (r = 0.672, *p* = 0.039), while it was negatively correlated with M0 Macrophages (r = −0.661, *p* = 0.044). Meanwhile, the immune infiltration analysis (Figure 7E) indicated that ESR1 had a positive correlation with CD4+ memory T cells (r = 0.87, *p* = 0.0027) and NK cells (r = 0.77, *p* = 0.014), but a negative correlation with regulatory T cells (r = −0.79, *p* = 0.0098), eosinophils (r = −0.83, *p* = 0.0056), and M0 macrophages (r = −0.89, *p* = 0.0013). Using the CIBERSORT method, we evaluated the immune infiltration features between SLE patients and controls. The bar plot depicted the relative percent of diverse immune cell subsets in each sample (Figure 7F). These results revealed that SIRT1 and ESR1 may be crucial to the progression in SLE.

## Discussion

The expression profiles of non-coding RNAs (ncRNAs), which include miRNAs, long non-coding RNAs (lncRNAs), and circular RNAs (circRNAs), have attracted increasing attention in various diseases due to the rapid growth of bioinformatics (26, 27). Compared with healthy individuals, most circRNAs were found to be abundant in peripheral blood, serum, T cells, peripheral blood mononuclear cells and kidney tissue, indicating their possible roles. In summary, circRNA dysregulation may have implications for key molecular processes involved in the pathogenesis of systemic lupus erythematosus (28). Therefore, a more comprehensive understanding of the importance of circRNAs in SLE is required. We used microarray data analysis to construct the circRNA-miRNA-mRNA network. The mRNAs in the network were used to identify significantly altered GO terms and the KEGG signaling pathway. The hub genes were then identified using the PPI network. In order to open up a new perspective on the therapy of SLE, certain effective substances have also been discovered.

Based on the results of functional enrichment analysis, we hypothesized that in SLE, the aberrant circRNAs utilize protein and RNA binding to regulate chromosome organization, chromatin organization, and negatively regulate adipocyte differentiation, as well as positively regulate colorectal migration of blood vessels. According to KEGG signaling pathway analysis, it is linked to cancer-related signaling pathways. Since the immunological and genetic pathways of SLE pathogenesis and the use of immunosuppressive drugs (ISDs) in treatment may increase the likelihood of such changes, the relevant literature has shown (29) that the risk of malignancy in SLE is considerable. In KEGG pathway analysis, it can be found that ESR1 is mainly related to endocrine resistance, the cancer signaling pathway, the estrogen signaling pathway, and Epstein-Barr virus infection, while SIRT1 is mainly related to the FoxO signaling pathway and Salmonella infection. Salmonella infection disrupts SIRT1/AMPK checkpoint control of mTOR to impair autophagy (30), and alterations of autophagy contribute to the progression of various autoimmune diseases, including systemic lupus erythematosus (SLE) (31). In the FoxO signaling pathway, such observations with Foxo3a in helper T cells imply disease relevance in SLE (32). However, the SIRT1-regulated signaling pathway of these hub genes (ESR1) in autoimmune diseases has not been reported. Whether it is related to the pathogenesis of SLE needs to be confirmed by further experiments.

SLE is more prevalent in women of reproductive age between the ages of 15 and 40, with a male-to-female incidence ratio of 1: 9. As a result, it has been suggested that estrogen may play a role in the development of SLE. In lymphoid nuclei, estrogen primarily interacts with estrogen receptor types 1 and 2 (ESR1 and ESR2, respectively) to carry out biological actions (33). Further evidence that ESR1, not ESR2, produces the lupus phenotype can be found in male mice lacking functioning ESR1. These mice are resistant to developing the lupus phenotype (34). Recent studies demonstrating that ESR1 promotes SLE in F1-generation female mice of the lupus mouse model (35) lend weight to this idea. In a previous investigation, Lee et al. (36) found that ESR1 gene polymorphisms were related to the age at which SLE first manifested in Korean patients; however, this study only included female participants. ESR1 rs2234693 and rs9340799 were revealed to be strongly linked with SLE susceptibility by Wang et al. (37). When Zhou et al. (38) compared the frequencies of C allele rs2234693 and G allele rs9340799 in the ESR1 gene between SLE patients and non-SLE controls, they found that the frequency of the C allele was considerably higher. Faslodex, an ESR1 antagonist, significantly decreased SLEDAI scores in SLE patients in a limited clinical trial (39). These findings support the idea that estrogen plays a part in the development of SLE.

SIRT1 is an NAD^+^-dependent monomeric protein that plays a significant role in important cellular activities, including cell differentiation, apoptosis, metabolism, aging, and immunological response. In Consiglio’s research, the SIRT1 promoter variant rs3758391 was found to be connected with SLE incidence, and the rs3758391 T allele may be linked to higher SLEDAI and lupus nephritis scores (40). Anti-dsDNA antibodies are well documented to fluctuate with SLE activity and to be closely associated with the symptoms of severe lupus (41, 42). Olivares et al. found that serum anti-dsDNA antibody levels in LN patients were significantly correlated with urinary SIRT1 mRNA levels, which is a valuable marker of renal injury (43). Systemic lupus erythematosus (SLE) patients had higher levels of the plasma protein SIRT1 (SIRT1) compared to healthy controls, and there was a significant correlation between the plasma SIRT1 concentration and disease activity, according to research by Yang et al. (44).

The final circRNA-miRNA-mRNA network contained HSA circ 0000345 and HSA miR-22-3p, which we identified. Only pertinent literature has demonstrated that there is currently no relationship between this circRNA and autoimmune disorders (45, 46). By suppressing HSA circ 0000345, HSA circ 0000345 plays a critical role in the treatment of atherosclerosis. The involvement of HSA circ 0000345 in endothelial cell damage is strongly related to the onset and progression of atherosclerosis. Down-regulation of HSA circ 0000345 has been demonstrated in the literature (47) to have an anti-tumor impact. However, there are no studies indicating that abnormal expression of HSA circ 0000345 contributes to the development of SLE. Future studies should verify the expression status of HSA circ 0000345 in SLE patients and its function. Additionally, Previous studies have demonstrated that miR-22-3p expression levels are increased in the peripheral blood of patients with lupus (48, 49), which is consistent with our findings. In contrast, compared to controls, SLE with lupus nephritis (LN) patients exhibited a downregulation of miR-22-3p (50). The disparate results may be attributed to the varying cell subtypes examined. Further experimentation is required to confirm its status and function.

Nevertheless, it is necessary to acknowledge the limitations of this work. First, the sample size of bone marrow for high-throughput sequencing was limited due to the difficulty in accessing bone marrow specimens from SLE patients and limited research funding. Additionally, the candidate genes screened were not validated with a large enough sample size, which resulted in a lack of rigor and precision. To address these issues, we plan to expand the sample size and validate the function and mechanism of action of the hub genes in SLE in further experiments.

## Conclusion

In summary, a novel circRNA-miRNA-mRNA ceRNA network (circ 0000345- miR-22-3-P-ESR1/SIRT1) was identified in SLE by bioinformatics and comprehensive analysis, which could be served as a possible biomarker or therapeutic target for SLE.

## Data availability statement

The original contributions presented in the study are publicly available. This data can be found here: GEO, accession GSE266651, https://www.ncbi.nlm.nih.gov/geo/query/acc.cgi?acc=GSE266651.

## Ethics statement

The studies involving humans were approved by the Ethics Committee of Fujian Provincial Hospital (Approval ID: K-2021-11-019). The studies were conducted in accordance with the local legislation and institutional requirements. The participants provided their written informed consent to participate in this study.

## Author contributions

JH: Writing – original draft. YD: Software, Visualization, Writing – original draft. JL: Data curation, Investigation, Writing – review & editing. HL: Writing – review & editing. FG: Methodology, Supervision, Writing – review & editing. ZC: Funding acquisition, Investigation, Methodology, Software, Visualization, Writing – review & editing. YW: Conceptualization, Data curation, Investigation, Software, Visualization, Writing – review & editing.

## Footnotes

^1^ http://www.ncbi.nlm.nih.gov/geo

^2^ http://geneontology.org/page/download-ontology

^3^ http://string-db.org/

^4^ http://software.broadinstitute.org/gsea/index.jsp

^5^ http://www.gsea-msigdb.org/gsea/downloads.jsp
